# Volumetric but Not Time Capnography Detects Ventilation/Perfusion Mismatch in Injured Rabbit Lung

**DOI:** 10.3389/fphys.2018.01805

**Published:** 2018-12-12

**Authors:** József Tolnai, Gergely H. Fodor, Barna Babik, André Dos Santos Rocha, Sam Bayat, Ferenc Peták, Walid Habre

**Affiliations:** ^1^Department of Medical Physics and Informatics, University of Szeged, Szeged, Hungary; ^2^Unit for Anesthesiological Investigations, Department of Anesthesiology, Pharmacology and Intensive Care, University of Geneva, Geneva, Switzerland; ^3^Department of Anesthesiology and Intensive Therapy, University of Szeged, Szeged, Hungary; ^4^EA-7442 RSRM Laboratory, Department of Clinical Physiology, Sleep and Exercise, Grenoble University Hospital, University of Grenoble, Grenoble, France

**Keywords:** mechanical ventilation, acute respiratory distress syndrome, respiratory monitoring, lung injury, ventilation to perfusion ratio, capnography

## Abstract

Whereas time capnography (Tcap) is routinely displayed during mechanical ventilation, the volumetric representation (Vcap) is seldom used. We compared the diagnostic value of indices derived from Tcap and Vcap following ventilation to perfusion ratio (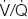) mismatch subsequent to experimentally induced acute respiratory distress syndrome (ARDS), and alveolar recruitment by elevating the positive end-expiratory pressure (PEEP). Lung injury was induced by iv lipopolysaccharide, whole lung lavage and injurious ventilation in anesthetized, mechanically ventilated rabbits (*n* = 26). Mainstream Tcap and Vcap were performed to assess normalized phase 2 (Sn2_T_, Sn2_V_) and phase 3 slopes (Sn3_T_, Sn3_V_) in the time and volumetric domains. Vcap was also used to estimate Enghoff’s physiological dead space (VD_E_). Lung oxygenation index (PaO_2_/FiO_2_) and intrapulmonary shunt (Qs/Qt) were derived from arterial and central venous blood gas samples. All measurements were made under baseline conditions, and, following lung injury, under moderate (6 cmH_2_O) and high PEEP levels (9 cmH_2_O). Lung injury deteriorated the PaO_2_/FiO_2_ (baseline vs. injured 466 ± 10.2 [95% confidence interval] vs. 77.3 ± 17.1 mmHg, *p* < 0.05) and compromised all mechanical parameters significantly, whereas Tcap parameters exhibited contradictory or inconsistent changes. Conversely, Vcap indices exhibited consistent changes and provided excellent diagnostic value in detecting lung-function deterioration subsequent to lung injury [area under the receiver operating characteristic (ROC) curve of 1.0 ± 0.0, 0.87 ± 0.22 and 0.86 ± 0.22 for VD_E_, Sn3_V_ and Sn3_V_/Sn2_V_, respectively]. Elevated PEEP increased PaO_2_/FiO_2_ and decreased Qs/Qt, which was reflected only in the Vcap slope ratio (Sn3_V_/Sn2_V_, *p* < 0.05). Our findings demonstrate the limited value of Tcap to detect ventilation to perfusion ratio (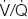) mismatch, following severe lung injury. Conversely, indices derived from Vcap proved to be sensitive for detecting lung volume loss and alveolar recruitment. Therefore, promotion of Vcap is of paramount importance as a real-time, non-invasive, bedside monitoring modality to detect the development of and to follow-up the progression of lung injury in a model of ARDS.

## Introduction

Clinicians are continuously challenged by the potentially deleterious pulmonary effects of mechanical ventilation. One of the characteristic manifestations of acute respiratory distress syndrome (ARDS) is increased microvascular permeability edema leading to extensive alveolar flooding, surfactant dysfunction and subsequent patchy atelectasis (Gattinoni et al., 2003; Chiumello et al., 2008). As a result, the matching of ventilation to perfusion (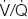) is locally deteriorated. Continuous, reliable and non-invasive monitoring of 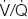 matching is of paramount importance in anesthesia and intensive care settings to titrate ventilation parameters, to detect ventilation-induced lung injury, and to guide ventilation strategies (Brochard et al., 2012; Theerawit et al., 2017). In this regard, capnography as a routinely applied continuous non-invasive bedside monitoring modality is gaining increasing attention, as it reflects changes in the homogeneity of alveolar ventilation and 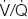 matching (Verscheure et al., 2016; Lam et al., 2017).

In routine clinical practice, the vast majority of the currently used anesthesia machines and intensive care ventilators display the expired CO_2_ concentration with respect to time (time capnography, Tcap). The slopes and angles as shape factors derived from Tcap are thought to reflect the dynamics of lung emptying and 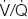 matching (Babik et al., 2012; Nassar and Schmidt, 2016). More relevant information can be extracted from the volumetric representation of the capnogram by plotting the expired CO_2_ concentration with respect to the expired gas volume (volumetric capnography, Vcap) (Suarez-Sipmann et al., 2014; Verscheure et al., 2016). In addition to the volumetric shape factors, Vcap also allows estimation of anatomical and physiological dead space parameters. If the latter is determined according to Enghoff’s definition (VD_E_) (Enghoff, 1938), both aspects of 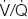 mismatch (dead space and intrapulmonary shunt) can be inferred (Tusman et al., 2011; Suarez-Sipmann et al., 2014; Bhalla et al., 2015; Verscheure et al., 2016).

The ability of Vcap to reflect the severity of ARDS and to assess lung recruitment subsequent to elevations in positive end-expiratory pressure (PEEP) is under debate. Whereas VD_E_ was shown to have prognostic value for detecting lung recruitment in ARDS (Suter et al., 1975; Nuckton et al., 2002), other studies did not detect consistent changes in Vcap dead space indices with respect to PEEP elevations in the presence of ARDS (Blanch et al., 1999; Beydon et al., 2002). An effect of PEEP on dead space parameters was detected only when concomitant increases in driving pressure occurred (Gagnon et al., 2002). Finally, the slope of phase 3 in Vcap (S3_V_) was shown to reflect alveolar recruitment in injured lungs (Tusman et al., 2011), whereas others found no relationship between PEEP elevations and changes in S3_V_ in both healthy patients and in those with ARDS (Blanch et al., 1999). Therefore, we aimed at comparing Tcap and Vcap parameters to detect dead space and intrapulmonary shunt causing 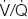 mismatch in an experimental model of ARDS. We also evaluated the ability of capnogram dead space and shape-factor parameters to reveal alveolar recruitment subsequent to PEEP elevations in injured lungs, as this evaluation is potentially important for the optimization of ventilation strategy.

## Materials and Methods

### Ethics

The experimental protocol was approved by the Experimental Ethics Committee of the University of Geneva and the Animal Welfare Committee of the Canton of Geneva, Switzerland (No. GE/94/15, 27 August 2015). All procedures were performed according to the current animal protection laws of Switzerland (LPA, RS455) and reported in compliance with ARRIVE guidelines.

### Animal Preparations

Twenty-six adult New Zealand White rabbits (3.04 ± 0.17 kg, 14 female, 12 male) were sedated with an intramuscular injection of xylazine (5.0 mg/kg) and general anesthesia was initiated and maintained with continuous intravenous infusion of propofol (15–20 mg/kg/h) and fentanyl (5 μg/kg/h) through a 22 G catheter in a marginal ear vein. The animals were tracheostomized following local lidocaine infiltration and were intubated with a 3.0 mm uncuffed endotracheal (ET) tube. Mechanical ventilation was initiated and maintained with a tidal volume (V_T_) of 7 ml/kg, a respiratory rate to achieve an end-tidal CO_2_ (ETCO_2_) of 5.5–6% (∼50/min), an inspired oxygen fraction (FiO_2_) of 0.4 and a PEEP of 6 cmH_2_O using the pressure control mode of a pediatric ventilator (Servo-i, Maquet Critical Care, Solna, Sweden). After the onset of proper depth of anesthesia was confirmed, neuromuscular blockade was initiated by continuous infusion of atracurium (0.6 mg/kg/h). Fluid balance of the animals was ensured by continuous infusion of lactated Ringer’s solution (4 ml/kg/h).

Invasive blood pressure monitoring and arterial blood gas analysis were performed via a 22 G catheter placed in the left femoral artery (Abbocath, Abbot Medical, Baar/Zug, Switzerland). Central venous blood gas samples were taken through a 16 G catheter placed in the right jugular vein (Arrow, Teleflex Medical Europe, Westmeath, Ireland). The electrocardiogram was monitored using subcutaneous needle electrodes. The animals were placed on a thermostatic heating pad and internal body temperature was continuously monitored and maintained at 38–39°C (Harvard Apparatus, South Natick, MA, United States). Arterial pressure and electrocardiogram were digitized at a sampling rate of 1 kHz and subsequently recorded (Powerlab model 8/35, ADInstruments, Dunedin, New Zealand).

### Measurement of Respiratory Mechanics

The airway and respiratory tissue mechanical parameters were measured by using the wave-tube method of the forced oscillation technique, as detailed previously (Bayat et al., 2009). Briefly, a loudspeaker-in-box system was used to generate a small-amplitude (1 cmH_2_O peak-to-peak) pseudorandom forcing signal at a frequency range of 0.5–20.75 Hz. This forcing signal was introduced through a polyethylene wave-tube (100 cm length, 0.375 cm internal diameter) into the tracheal cannula during short (8 s) end-expiratory apneas. To maintain constant pressure throughout the recordings, the loudspeaker chamber and the wave-tube were pressurized to the level of PEEP. Lateral pressures were measured at the loudspeaker (P_1_) and the tracheal end (P_2_) of the wave-tube using miniature pressure transducers (ICS 33NA00D, Milpitas, CA, United States). The pressure signals were low-pass filtered at 25 Hz corner frequency and digitized at 256 Hz by an analog-digital converter board (USB-6211, National Instruments, Austin, TX, United States). From the pressure transfer function (P_1_/P_2_) calculated by fast Fourier transformation, the input impedance of the respiratory system (Zrs) was derived. Three to four comparable 8-s recordings were taken during each time point, and the Zrs spectra were averaged for further processing.

Airway and tissue mechanical properties were calculated from the Zrs spectra by model fitting (Hantos et al., 1992) with the help of a global optimization method. The well validated model consists of airway resistance (Raw) and airway inertance (Iaw) in series with a constant-phase tissue compartment including tissue damping (G) and tissue elastance (H). As previously established, Raw reflects mainly the flow resistance of the conducting airways, Iaw is related to the cyclic acceleration and deceleration of the intra-thoracic gas, G describes the energy loss within the respiratory tissues (resistance), and H characterizes the energy storage capacity of the respiratory tissues (elastance). All Zrs spectra were corrected for the instrumental components of the ET tube and the tubing of the circuit by subtracting them before model fitting.

### Recording and Analyses of Time and Volumetric Capnograms

Ventilation airflow and changes in CO_2_ concentration were recorded by a pediatric-size Y-piece flow sensor and the mainstream capnometer of the Servo-i ventilator. These signals were digitized by a computer through a serial port connection at a sample rate of 100 Hz.

Time series of the expiratory capnogram data were fitted by using the Levenberg-Marquardt optimization algorithm to eliminate high-frequency noise (Tusman et al., 2009), and thereby, allow more accurate identification of shape factors and dead-space parameters. Vcap curves were generated from the fitted time capnograms and the simultaneously recorded volume signals derived from the recorded airflow data by integration.

The entire expiratory phase of each breathing cycle was identified from the driving signal of the expiratory valve of the respirator. Identification of the capnogram phases and calculation of shape factors both in the time and volumetric domains were based on previously established methods (Tusman et al., 2009). The inflection point of phase 2 was localized as the maximum of the first derivative of the capnogram curve in both the time and volumetric domains. The maximum of the third derivative of the Tcap and Vcap before and after this inflection point were also identified and this range was considered capnogram phase 2, which reflects mixed emptying of airway-alveolar spaces. Phase 3 of the capnogram curves, which represents expiration of the alveolar gas compartment, is defined as the range from the end of phase 2 until the end of expiration.

Phase 2 slopes in the time (S2_T_) and volumetric (S2_V_) domains were derived by fitting a linear regression line to three points around the inflection point. Phase 3 slopes of the time (S3_T_) and volumetric (S3_V_) capnograms were calculated by fitting a linear regression line to the middle-third of the phase 3 sections. Induction of ARDS led to marked decreases in ETCO_2_; this factor was compensated by dividing these slope indices by the corresponding ETCO_2_ values, thereby obtaining normalized time (Sn2_T_, Sn3_T_) and volumetric (Sn2_V_, Sn3_V_) phase 2 and phase 3 slopes.

Vcap curves also allow assessment of dead space indices. Fowler’s anatomic dead space (VD_F_) was defined as the expired gas volume until the inflection point in phase 2 (Fowler, 1948). Bohr’s physiological dead space (VD_B_) was calculated as (Bohr, 1891):

$$VD_{\text{B}}/V_{\text{T}}=(PA{\text{CO}}_{\text{2}}-P\bar{E}{\text{CO}}_{\text{2}})/PA{\text{CO}}_{\text{2}}$$

where P_ACO2_ is the mean alveolar partial pressure of CO_2_ determined as CO_2_ concentration at the midpoint of phase 3 in the Vcap curve. ${\text{P}}_{\bar{E}\text{CO2}}$ is the mixed expired CO_2_ partial pressure obtained by dividing the integrated Vcap curve by V_T_ in each expiratory cycle.

Enghoff’s modified physiological dead space (VD_E_), which also includes the intrapulmonary shunt (not ventilated but perfused alveoli), was calculated as (Enghoff, 1938):

$$VD_{\text{E}}/V_{\text{T}}=(Pa{\text{CO}}_{\text{2}}-P\bar{E}{\text{CO}}_{\text{2}})/Pa{\text{CO}}_{\text{2}}$$

where PaCO_2_ is the partial pressure of CO_2_ in the arterial blood sample.

Differences between Enghoff’s and Bohr’s dead spaces (VD_E_ – VD_B_) were also determined. This difference represents the intrapulmonary shunt circulation (i.e., the virtual lung volume corresponding to the perfused, but not ventilated, alveoli).

### Blood Gas Analyses

Blood gas samples were analyzed by a point-of-care blood gas analyzer (i-Stat, Abbott Laboratories, Chicago, IL, United States). Oxygen partial pressures (PaO_2_ and PvO_2_), carbon-dioxide partial pressures (PaCO_2_ and PvCO_2_) and oxygen saturation (SaO_2_ and SvO_2_) were measured in arterial and venous blood samples. Hemoglobin content (Hb) was determined by a veterinary blood count analyzer (Sysmex pocH-100i, Sysmex Corporation, Kobe, Japan).

The intrapulmonary shunt fraction (Qs/Qt) was calculated with the Berggren equation (Berggren, 1942):

$$Qs/Qt=(CcO_{\text{2}}-CaO_{\text{2}})/(CcO_{2}-CvO_{2})$$

where CaO_2_, CvO_2_, and CcO_2_ are the arterial, central venous and pulmonary capillary blood oxygen contents, respectively. CcO_2_ was derived from the alveolar gas equation with the assumption that hemoglobin in the pulmonary capillaries was 100% saturated:

(1) $$CcO_{2}=1.34ml/g\times Hb+Sol\times (FiO_{2}\times 713mmHg-PaCO_{2}/0.8)$$

where 1.34 ml/g is Hüfner’s constant, Sol is 0.0031 ml/100 ml/mmHg, 713 mmHg is the total dry gas pressure and 0.8 is the respiratory exchange ratio.

### Study Protocol

Following a 10-min period allowed for the stabilization of vital parameters, baseline measurements were performed by registering Tcap and Vcap data, recording Zrs spectra and analyzing arterial and venous blood gas samples. Lung injury (as a model of ARDS) was then induced with a multi-hit model including a combination of 300 μg/kg intravenous lipopolysaccharide (*Escherichia coli* O111:B4, Sigma, St. Louis, MO, United States), repeated whole lung lavage (injecting and removing 5 × 60 ml 30°C normal saline through the ET tube) and injurious ventilation (0 cmH_2_O PEEP, V_T_ = 10 ml/kg and FiO_2_ = 1, 20–30 min). This treatment ensured the development of severe ARDS characterized by a PaO_2_/FiO_2_ ratio of approximately 100, according to the Berlin definition (ARDS Definition Task Force Ranieri et al., 2012). FiO_2_ was then decreased to 0.9 and the animals were randomly divided into two groups: rabbits were either ventilated at a PEEP of 6 cmH_2_O (*n* = 13) or 9 cmH_2_O (*n* = 13). Following another 10-min stabilization period, capnogram, Zrs-spectra and blood gas parameter recordings were repeated.

### Statistical Analyses

Data are presented as mean ± standard deviation (SD). Two-way repeated measure analyses of variances (ANOVA) with Holm-Sidak *post hoc* tests were carried out to assess the effects of lung injury (baseline vs. injured) and the effects of PEEP (6 vs. 9 cmH_2_O) on respiratory mechanical, capnography-derived and blood-gas parameters. To assess the ability of capnography and respiratory mechanical parameters to detect lung-function deterioration, receiver operating characteristic (ROC) curves were analyzed using the anticipated changes of each parameter value (increases in phase 3 slopes and decreases in phase 2 slopes and dead space indices; You et al., 1994; Blanch et al., 1999; Chan et al., 2017) as identifying criteria. From the ROC analyses, the area under the curve (AUC) was calculated with 95% confidence intervals (CIs). If the 95% CI of an AUC contained 0.5, the variable was considered an uninformative classifier. The sample size was estimated for a 2-way repeated measures ANOVA on Sn_3V_ as the main outcome variable with an expected effect size of 0.8, a power of 0.8 and 2-sided alpha error of 0.05 (Bausell, 2002). The estimation resulted in a required sample size of 13 for each group. All statistical tests were carried out with the SigmaPlot software (version 13, Systat Software, Inc., Chicago, IL, United States), using a significance level of *p* = 0.05; all *p*-values were two-sided.

## Results

**FIGURE 1:**
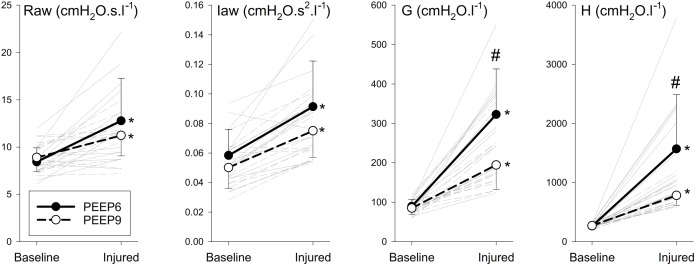
Mechanical parameters of the respiratory system before and after lung injury. Thick lines denote group averages (±SD), and thin lines denote changes for individual rabbits. Raw, airway resistance; Iaw, airway inertance; G, tissue damping; H, tissue elastance. ^∗^*p* < 0.05 baseline vs. injured; ^#^*p* < 0.05 PEEP6 vs. PEEP9.

Mechanical parameters of the respiratory system before and after lung injury are shown in Figure 1. At both PEEP levels, significant elevations were observed for Raw, Iaw, G and H in the injured lungs (*p* < 0.001 for all). No significant interaction between PEEP and lung injury was evidenced for the airway parameters, demonstrating that the level of PEEP did not significantly affect deterioration in airway mechanics. However, significant interactions between the PEEP and lung injury were observed for G (*p* < 0.001) and H (*p* < 0.005), evidencing the protective effect of PEEP against elevations in tissue viscoelastic indices. These significant interactions were manifested in the significantly greater values for G (*p* < 0.001) and H (*p* < 0.001) when the lungs were ventilated with the lower PEEP.

**FIGURE 2:**
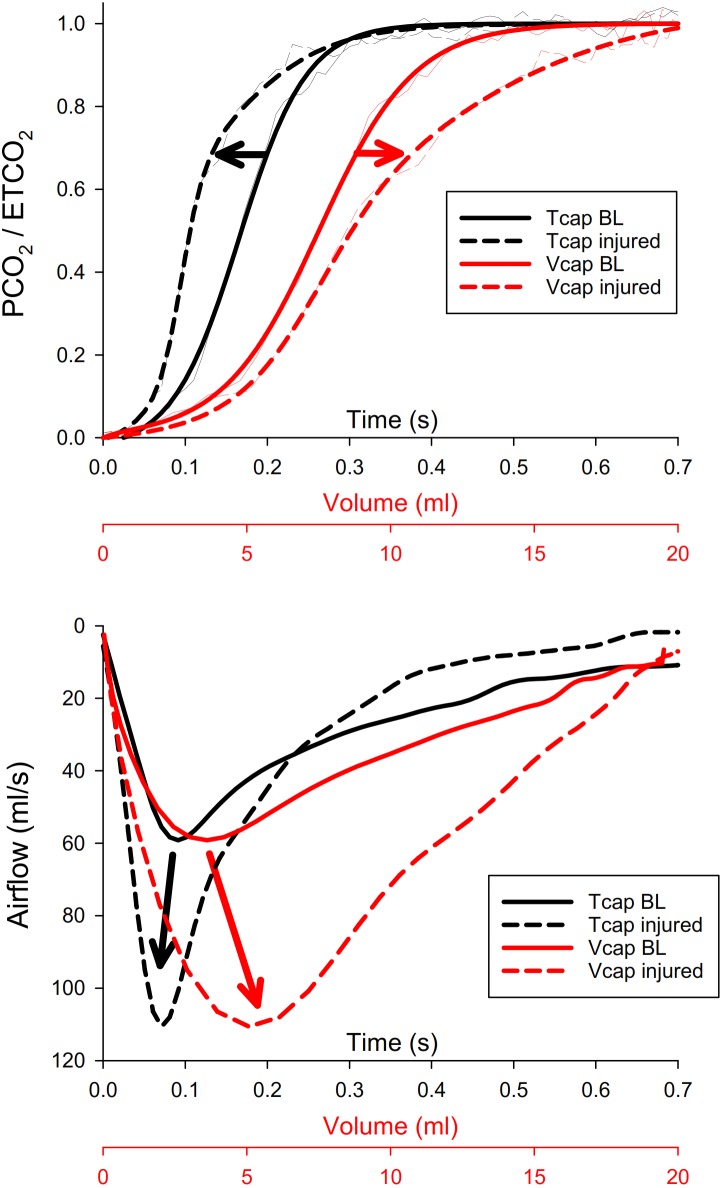
Top panel: normalized time (Tcap, black) and volumetric capnogram (Vcap, red) curves obtained in a representative animal with healthy lungs (BL, solid lines) and after inducing lung injury (injured, dashed lines). Thin lines denote the original curves, whereas thick curves are fitted with a Levenberg-Marquardt algorithm. Phase 2 of Vcap and Tcap is the rapidly ascending part of the sigmoid-shape curve, while phase 3 is the upper flattened part of the sigmoid curve. PCO_2_, partial carbon-dioxide pressure; ETCO_2_, end-tidal partial pressure of carbon-dioxide. Bottom panel: corresponding airflow curves under the same conditions as in the top panel, both as a function of time (Tcap, black) and of volume (Vcap, red). Curves are synchronized with those in the top panel.

Normalized Tcap and Vcap curves obtained in a representative animal with healthy lungs and after inducing lung injury and the corresponding changes in airflow as a function of time or expired volume are shown in Figure 2. In the Tcap curves, slight elevation in the phase 2 slope (i.e., the rapidly ascending parts of the sigmoid-shape curves) and no obvious change in the phase 3 slope (i.e., the upper flattened parts of the sigmoid curves) were detected after lung injury. This makes the Tcap curve more “square-like” in the injured lung. The top panel also demonstrates that Vcap exhibits opposite changes, with a decreased phase 2 slope and increased phase 3 slope after lung injury compared to BL, making the Vcap curve more “shark fin-like” following lung injury.

The lower panel demonstrates the opposite shifts in the peak expiratory airflow after lung injury: in the time domain, lung injury shifted the increased expiratory peak flow to the earlier phase of expirations. However, when the expiratory flow was plotted against the expired volume, it was observed that ARDS shifted the increased expiratory peak flow to the right, demonstrating a delay in the volumetric domain.

**FIGURE 3:**
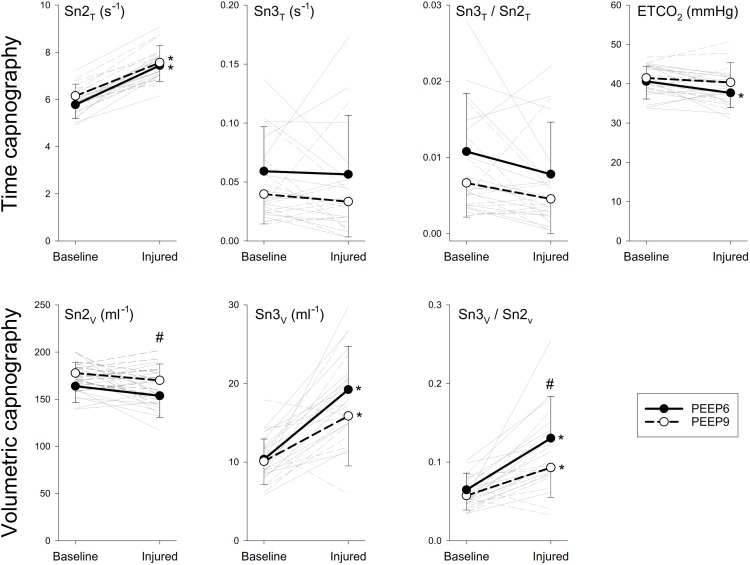
Time (Tcap) and volumetric capnography (Vcap) shape factors before and after lung injury. Thick lines denote group averages (±SD), whereas thin lines denote changes observed for individual animals. Sn2_T_ and Sn2_V_, normalized phase 2 slope in the time and volumetric capnogram, respectively; Sn3_T_ and Sn3, normalized phase 3 slope of the time and volumetric capnogram, respectively; ETCO_2_, end-tidal partial pressure of carbon-dioxide. ^∗^*p* < 0.05 baseline vs. injured; ^#^*p* < 0.05 PEEP6 vs. PEEP9.

Figure 3 demonstrates normalized Tcap and Vcap shape factors representing the phase 2 and phase 3 slopes and their ratios. Among the slope indices obtained from Tcap, only Sn2_T_ exhibited slight but statistically significant changes after lung injury (*p* < 0.001), and none of the parameters differed for the PEEP changes. However, marked and statistically significant elevations were observed in the normalized third phase slope of the Vcap curves (Sn3_V_, *p* < 0.001). These changes were also manifested in the significantly higher values of the Sn3_V_/Sn2_V_ slope ratio (*p* < 0.001), and this volumetric index also demonstrated significant differences for different PEEP levels after injury (*p* < 0.02).

**FIGURE 4:**
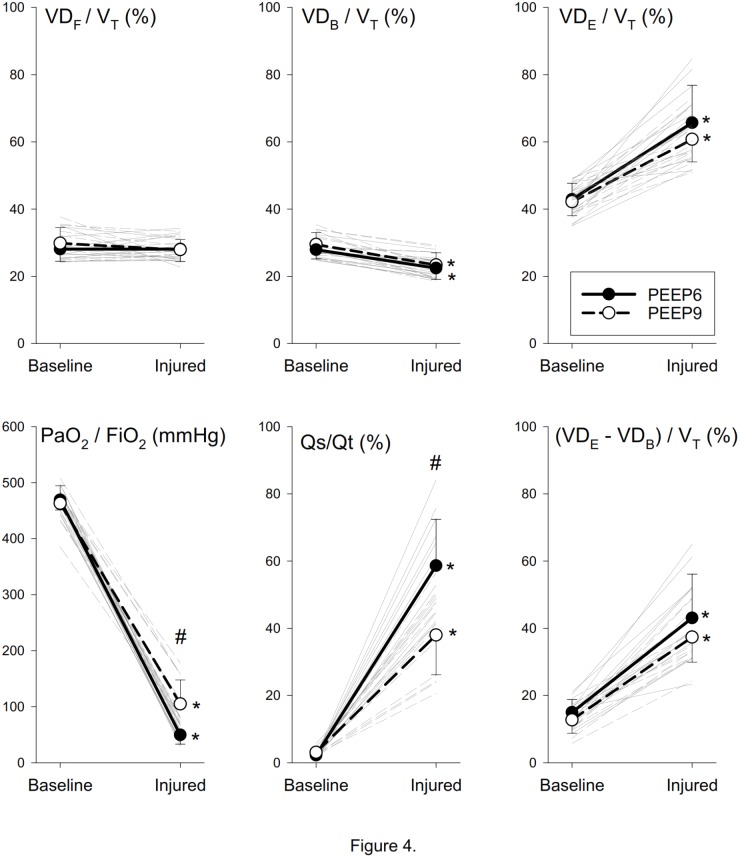
Normalized dead space, oxygenation and shunt parameters before and after lung injury. Thick lines denote group averages (±SD), whereas thin lines denote changes observed for individual animals. VD_F_, anatomical dead space according to Fowler’s definition; V_T_, tidal volume; VD_B_, physiological dead space according to Bohr’s definition; VD_E_, physiological dead space according to Enghoff’s definition; PaO_2,_ arterial partial pressure of oxygen; FiO_2_, fraction of inspired oxygen; Qs/Qt, intrapulmonary shunt fraction. ^∗^*p* < 0.05 baseline vs. injured; ^#^*p* < 0.05 PEEP6 vs. PEEP9.

Anatomical (VD_F_) and physiological (VD_B_, VD_E_) dead space parameters normalized to V_T_, PaO_2_/FiO_2_ and parameters reflecting intrapulmonary shunt are depicted in Figure 4. Neither lung injury nor PEEP had significant effect on the VD_F_/V_T_. VD_B_/V_T_ exhibited slight but statistically significant decreases in the injured lungs (*p* < 0.001); whereas marked elevations were observed in VD_E_/V_T_ (*p* < 0.001). The significantly compromised lung oxygenation index was associated with marked elevations in intrapulmonary shunt, both when expressed as Qs/Qt (*p* < 0.001) and as VD_E_ – VD_B_ (*p* < 0.001). Elevation in PEEP improved the lung oxygenation index (*p* < 0.001), which was reflected in a lower Qs/Qt (*p* < 0.001).

**FIGURE 5:**
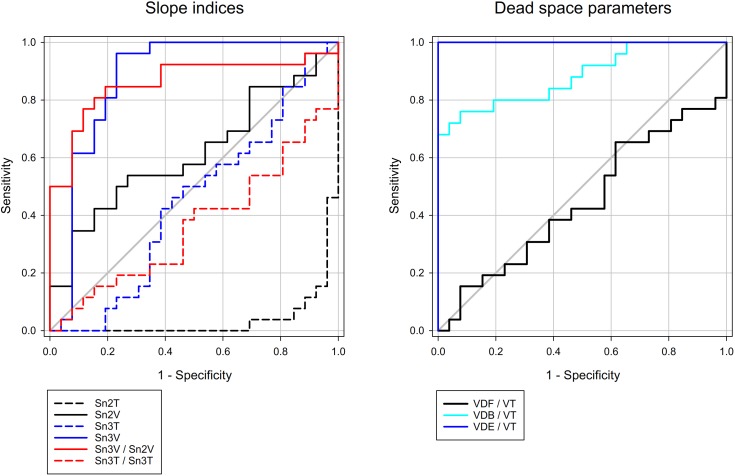
Receiver operating characteristic curves of selected capnogram shape factors and dead space parameters. Corresponding AUC values with 95% CI can be found in Table 1. Diagonal gray line indicates AUC of 0.5 (no-discrimination line).

Table 1 summarizes the area under the ROC curves for each forced oscillatory, Tcap, and Vcap parameter. All respiratory mechanical parameters determined by forced oscillations exhibited high AUC values. None of the Tcap parameters exhibited AUC values that were sufficiently high to discriminate lung injury. In contrast, Sn3_V_, the Sn3_V_/Sn2_V_ slope ratio, VD_B_/V_T_, and VD_E_/V_T_ exhibited excellent AUC values, demonstrating these indices can be used for the detection of lung injury. The ROC curves for the most interesting parameters are also displayed in Figure 5.

**Table 1:** Area under the ROC curves and their 95% confidence intervals (95% CI).

Measurement	Parameters	ROC curve area (AUC)	95% CI
FOT	Raw	0.82	0.71–0.94
	Iaw	0.85	0.75–0.95
	G	1.0	1.00–1.00
	H	1.0	1.00–1.00
Tcap slopes	Sn2_T_	0.04	0.00–0.08
	Sn3_T_	0.45	0.29–0.61
	Sn3_T_/Sn2_T_	0.37	0.21–0.52
Vcap slopes	Sn2_V_	0.39	0.23–0.54
	Sn3_V_	0.87	0.76–0.98
	Sn3_V_/Sn2_V_	0.86	0.75–0.97
Vcap dead space	VD_F_/V_T_	0.45	0.29–0.61
	VD_B_/V_T_	0.88	0.79–0.88
	VD_E_/V_T_	1.00	1.00–1.00
Intrapulmonary shunt	Qs/Qt	0.99	0.98–1.00

## Discussion

Shape factors and dead space parameters obtained from volumetric capnogram reflect ventilation/perfusion defects, however it has not been clarified whether Vcap is an appropriate tool to assess severity of ARDS and to detect lung recruitment. In the present study, fundamentally distinct changes were found in the shape of the time and volumetric capnograms in the present experimental model of lung injury. The normalized phase 3 slope of the time capnogram, which reflects alveolar emptying, exhibited inconsistent changes following the induction of ARDS and elevations in PEEP. In contrast, the phase 3 slope obtained from the volumetric capnogram consistently detected the loss of lung volume subsequent to lung injury, as did the changes observed in lung oxygenation and respiratory mechanics. Alveolar recruitment with PEEP in the injured lungs was reflected in the ratio of normalized slopes of the second and third phases of the volumetric capnogram. ROC curve analysis confirmed that only volumetric capnography parameters and dead space indices could discriminate healthy and injured lungs; whereas time capnography indices were unable to do so.

### Time Capnography

Tcap is part of routine patient monitoring during mechanical ventilation both for anesthesia and in intensive care settings. Whereas Tcap does not allow the determination of dead space parameters, the shape factors describing the slopes of phases 2 (S2_T_) and 3 (S3_T_) and their angles have been found to characterize 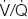 matching (Babik et al., 2012; Nassar and Schmidt, 2016). S3_T_, the most commonly used shape factor derived from Tcap, represents the heterogeneity of lung perfusion and ventilation (Yuda Sutherasan Lrenzo Ball Pelosi, 2015). Heterogeneity of lung ventilation explains why increases in Raw have been associated with elevated S3_T_ in mechanically ventilated patients (Babik et al., 2012; Chan et al., 2017), as well as the close relationships between S3_T_ and the severity of airway obstruction in asthmatic subjects (You et al., 1994; Yaron et al., 1996; Nik Hisamuddin et al., 2009).

Unlike previous studies, in which airway obstruction was the primary cause of lung function deterioration, changes in Sn3_T_ observed in the present study were inconsistent for injured lung (Figure 3). This discrepancy may be explained by the fact that the mechanical abnormality in ARDS lung was primarily reduced lung compliance and to a lesser extent, increased airway resistance. Specifically, the accelerating impact of the elevated lung recoil outweighed the decelerating effects of the increased Raw on regional alveolar emptying (Babik et al., 2012; Chan et al., 2017).

### Volumetric Capnography

In the absence of severe bronchoconstriction leading to decreases in gas volume in the conducting airways, no evidence for a change in VD_F_ was observed in the presence of ARDS (Figure 4). In addition, VD_B_ contains the gas volume of the ventilated but not perfused alveolar compartments (Bohr, 1891). Accordingly, the uniform decreases and the high diagnostic value of VD_B_ (Figure 5) may be attributed to the hypoxic pulmonary vasoconstriction that was likely to develop in the presence of severely compromised lung oxygenation index (PaO_2_/FiO_2_) in the injured lungs. As an extension of VD_B_, VD_E_ reflects physiological dead space resulting from all types of 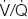 mismatch, including the intrapulmonary shunt (Enghoff, 1938). Accordingly, the difference between VD_E_ and VD_B_ is related to a virtual dead space volume of the shunted alveolar units. The strong and significant correlation (*r* = 0.81, *p* < 0.001) between VD_E_-VD_B_ and the classical shunt fraction (Qs/Qt) supports this concept and agrees with previous findings (Csorba et al., 2016).

Since Qs/Qt consistently increases in lung injury (Nirmalan et al., 2001), the uniform increases (Figure 4) and the perfect classification ability of VD_E_ (Figure 5) can be attributed to this feature. This finding agrees with previous results obtained in earlier studies demonstrating the ability of VD_E_ to detect both shunt and dead space component of 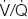 mismatch in ARDS (Suter et al., 1975 Blanch et al., 1999; Nuckton et al., 2002; Tusman et al., 2006).

Changes in the shape of Vcap provided relevant information with the development of 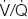 mismatch. Unlike Tcap, the transition of the normal Vcap toward a “shark fin” shape was consistently observed as a result of Sn2_V_ flattening and increasing steepness of Sn3_V_. These differences between the two modalities can be explained by the increased impedance of the respiratory system, which overexpresses the effect of compressible volume during mechanical ventilation (Neve et al., 2003). While rapid expansion of the exhaled compressible gas at the beginning of expiration may contribute to the steepening of Sn2_T_, this phenomenon does not bias the Vcap parameters, which is time-invariant and directly proportional to the expired gas volume. The same phenomenon may also explain the differences observed in Sn3_T_ and Sn3_V_ (Figure 3). This difference also contributes to the excellent diagnostic value of Sn3_V_ (Figure 5) for revealing the development of lung volume loss in lung injury, in accordance with previous findings reporting the sensitivity of S3_V_ to detect lung collapse (Tusman et al., 2006).

In the injured lungs, recruiting the alveoli by elevating PEEP led to an improvement in the lung oxygenation index and in Qs/Qt. The capnographic parameter used to detect this improvement was the Sn3_V_/Sn2_V_ slope ratio (Figure 5). These results further demonstrate the diagnostic value of Vcap in the detection of the recruitment of the alveolar units in an injured lung.

### Limitations

While measurements of Tcap, Vcap, respiratory mechanics and gas exchange could have been obtained in a clinical setting, we characterized the whole spectrum offered by the capnography under well-controlled experimental conditions in order to avoid the biasing effects of comorbidities leading to substantial interindividual variability in mechanically ventilated patients with ARDS. Despite the severity of ARDS induced by repeated lavage and injurious mechanical ventilation, the improvement obtained in the lung oxygenation index, intrapulmonary shunt and respiratory tissue mechanics after elevating PEEP confirms the relevance of the model as a method to compare the value of Tcap and Vcap. Furthermore, our current study is limited to characterizing the utility of Tcap and Vcap parameters in a population where the severity of lung injury is more homogeneous than that observed in a clinical setting. While VD_B_ and VD_E_ exhibit good classification abilities in the case of severely impaired respiratory function, full characterization of the dependence of these parameters on the severity of lung injury warrants further investigations.

## Conclusion

The present experimental investigation highlighted the limitations of time capnography as an on-line noninvasive bedside monitor to detect factors leading to the 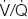 mismatch that develops in lung injury. Conversely, volumetric capnography revealed an excellent ability to detect uneven caused by both increased dead space and intrapulmonary shunt. Comparison of time and volumetric capnography techniques revealed that a dead-space index incorporating intrapulmonary shunting (Enghoff’s dead space), and the phase 3 slope were the most sensitive markers for atelectasis development and alveolar flooding, which are hallmark features of ARDS. The Sn3_V_/Sn2_V_ slope ratio, which expresses the combination of the flattened volumetric phase 2 slope and the steepened phase 3 slope, revealed 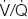 mismatch, as well as allowed detection of alveolar recruitment subsequent to PEEP elevation. Therefore, we conclude it is necessary to promote the implementation of volumetric capnography as a routine monitoring modality in clinical practice both in anesthesia and intensive care settings.

## Data Availability Statement

The raw data supporting the conclusions of this manuscript will be made available by the authors, without undue reservation, to any qualified researcher.

## Author Contributions

JT and FP performed experimental design, data analysis, interpretation of results, and drafting of the manuscript. GF performed measurement of data, data analysis, interpretation of results and drafting of the manuscript. BB, SB, and WH performed experimental design, interpretation of results, and drafting of the manuscript. ADSR performed measurement and analysis of the data. All authors have read, edited, and approved the final version of the manuscript and agreed to be accountable of all aspects of this work.

## Conflict of Interest Statement

The authors declare that the research was conducted in the absence of any commercial or financial relationships that could be construed as a potential conflict of interest.

## Abbreviations

ANOVA: analyses of variances
ARDS: acute respiratory distress syndrome
AUC: area under the curve
CaO_2_: oxygen content of arterial blood
CcO_2_: oxygen content of capillary blood
CI: confidence interval
CvO_2_: oxygen content of venous blood
ET: endotracheal
ETCO_2_: end-tidal partial pressure of carbon-dioxide
FiO_2_: fraction of inspired oxygen
G: tissue damping
H: tissue elastance
Hb: hemoglobin content
Iaw: airway inertance
P_1_: lateral pressure measured at the loudspeaker end of the wave-tube
P_2_: lateral pressure measured at the tracheal end of the wave-tube
PaCO_2_: arterial partial pressure of carbon-dioxide
P_ACO2_: mean alveolar partial pressure of carbon-dioxide
PaO_2_: arterial partial pressure of oxygen
mixed expired partial pressure of carbon-dioxide
PEEP: positive end-expiratory pressure
PvCO_2_: venous partial pressure of carbon-dioxide
PvO_2_: venous partial pressure of oxygen
Qs/Qt: intrapulmonary shunt fraction
Raw: airway resistance
ROC: receiver operating characteristic
S2_T_: second-phase slope of the time capnogram
S2_V_: second-phase slope of the volumetric capnogram
S3_T_: third-phase slope of the time capnogram
S3_V_: third-phase slope of the volumetric capnogram
SaO_2_: oxygen saturation of arterial blood
SD: standard deviation
Sn2_T_: normalized second-phase slope of the time capnogram
Sn2_V_: normalized second-phase slope of the volumetric capnogram
Sn3_T_: normalized third-phase slope of the time capnogram
Sn3_V_: normalized third-phase slope of the volumetric capnogram
SvO_2_: oxygen saturation of venous blood
Tcap: time capnography
Vcap: volumetric capnography
VD_B_: physiological dead space according to Bohr’s definition
VD_E_: physiological dead space according to Enghoff’s definition
VD_F_: anatomical dead space according to Fowler’s definition
ventilation to perfusion ratio
V_T_: tidal volume
Zrs: input impedance of the respiratory system
